# Analysis of ERCC1 and ERCC2 gene variants in osteosarcoma, colorectal and breast cancer

**DOI:** 10.3892/ol.2015.2894

**Published:** 2015-01-26

**Authors:** BENJAMÍN GÓMEZ-DÍAZ, MARÍA DE LA LUZ AYALA-MADRIGAL, MELVA GUTIÉRREZ-ANGULO, AURA ERAZO VALLE-SOLIS, LUIS MIGUEL LINARES-GONZÁLEZ, ROBERTO GONZÁLEZ-GUZMÁN, DAVID CRUZ-GUILLÉN, ANA LILIA CEDEÑO-GARCIDUEÑAS, PATRICIA CANTO, LUZ BERENICE LÓPEZ-HERNÁNDEZ

**Affiliations:** 1 National Institute of Rehabilitation, México City, México; 2 Institute of Human Genetics, UCHS, University of Guadalajara, Wet Saw, Guadalajara, México; 3 Department of Clinics, UCALTOS, University of Guadalajara, Tepatitlán, Jalisco, México; 4 National Medical Centre ‘20th November,’ Institute for Social Security of State Workers, México City, México; 5 Obesity Research Unit, Faculty of Medicine, National Autonomous University of Mexico, México City, México

**Keywords:** ERCC1, ERCC2, cancer, Mexican-mestizos

## Abstract

The Asn118Asn (rs11615) variant in the *ERCC1* gene, and the Lys751Gln (rs13181) and Asp312Asn (rs1799793) variants in the *ERCC2* gene have been associated with the development of varied types of cancer. The aim of the present study was to test for any association between the *ERCC1* and *ERCC2* gene variants and three different types of cancer in Mexican-mestizo patients. Patients and their respective controls were formed into three groups: The osteosarcoma group, with 28 patients and 97 controls; the colorectal group, with 108 patients and 119 controls; and the breast cancer group, with 71 patients and 74 controls. Genotyping was performed using TaqMan probes and quantitative polymerase chain reaction. Allele and genotype frequencies were compared using a χ^2^ test. Only one SNP (rs1799793) was found to be associated with breast cancer. This is the first study analyzing the SNPs in *ERCC1* and *ERCC2* genes and the susceptibility to cancer in Mexican-mestizo patients with osteosarcoma, and colorectal and breast cancer.

## Introduction

Excision repair cross-complimentary group 1 (ERCC1) and group 2 (ERCC2) are involved in DNA repair bringing stability and integrity to the genome (1). The *ERCC1* and *ERCC2* genes are part of the nucleotide excision repair pathway, which is usually indicated to be involved with repair adducts, oxidative DNA damage, cross links, alkylating damage and thymidine dimers (2,3). Therefore, the presence of single nucleotide polymorphisms (SNPs) in each gene has been associated with the development of varying types of cancer, depending on the exposure of specific environmental risk factors (4–12). In particular, the Asn118Asn (rs11615; c.354G>A) variant in *ERCC1* (13), and the Lys751Gln (rs13181; c.2251A>C) and Asp312Asn (rs1799793; c.934C>T) variants in *ERCC2* (11,12,14) have been associated with the risk of cancer in a variety of populations. However, the association with the Mexican-mestizo population has not been tested, even though ethnicity may play a crucial role for case-control based studies for genetic susceptibility variants in complex diseases (15). The aim of the present study was to test for an association between the Asn118Asn (*ERCC1*), Lys751Gln and Asp312Asn (*ERCC2*) variants, and osteosarcoma and colorectal and breast cancer in Mexican patients.

## Patients and methods

### Patient groups

Analyses were conducted for the following groups: The osteosarcoma group, with 28 patients and 97 healthy controls; the colorectal cancer group, with 108 patients and their respective 119 controls; and the breast cancer group, with 71 females diagnosed with breast cancer and 74 controls. All individuals were of Mexican-mestizo ethnicity. Institutional Committees approved the study and informed written consent was obtained from all participants. the institutions that approved the collection of samples were The National Institute of Rehabilitation, Mexico City, for samples of osteosarcoma tissue, The Civil Hospital of Guadalajara, Guadalajara, Jalisco, for samples of colon cancer tissue and The National Medical Center ‘20 of November’, Institute for Social Security and Services for State Workers, Mexico City, Mexico for samples of breast cancer tissue. Patients were recruited between May 2012 and December 2013, from three tertiary-care level hospitals in Mexico (National Institute of Rehabilitation, Mexico City; National Medical Center ‘20 of November’, Institute for Social Security and Services for State Workers, Mexico City; and Civil Hospital of Guadalajara, Jalisco, Mexico).

### Data collection

Samples of peripheral blood (300 μl) were obtained from patients and genomic DNA was isolated by the CTAB-DTAB method that relies on the properties of cationic detergents (16). Genotyping was performed by quantitative polymerase chain reaction (qPCR) using TaqMan probes (hydrolysis probes) and the C_2532959_1_ (rs11615/Asn118Asn *ERCC1*), C_3145033_10 (rs13181/Lys751Gln *ERCC2*) and C-3145050_10 (rs1799793/Asp312Asn *ERCC2*) assays (Applied Biosystems, Foster City, CA, USA). qPCR was performed on a LightCycler 480 Instrument II (Roche Diagnostics GmbH, Basel, Switzerland), according to the manufacturer’s instructions. Briefly, PCR reactions contained 10–20 ng of DNA, 5.0 μl of Maxima Probe qPCR Master Mix (2X; Thermo Fisher Scientific, Austin City, TX, USA), 0.25 μl of the primers and probes (10X) and H_2_O to 10 μl. PCR conditions were 95°C for 10 min, 55 cycles of amplification (95°C for 30 sec, 59°C for 30 sec and 72°C for 40 sec) and a final extension at 72°C for 5 min. Genotype assignment was performed with LightCycler 480 software version 1.5.0 (Roche Diagnostics GmbH).

### Statistical analysis

Statistical analysis was performed using SPSS software version 18.0 (SPSS, Inc., Chicago, IL, USA) and P<0.05 was considered to indicate a statistically significant difference. The allele and genotype frequencies, and the distribution of genotypes in groups of patients and controls were compared by χ^2^ or Fisher’s exact test. Homozygotes for the most frequent allele were used as reference groups. For measuring the association between genetic polymorphisms and the risk of cancer, odds ratios (ORs) were calculated. Hardy-Weinberg equilibrium (HWE) was estimated using the χ^2^ test (http://ihg.gsf.de/cgi-bin/hw/hwa1.pl; accessed 20/08/2013).

## Results

The clinical data of the patients with osteosarcoma, and colorectal and breast cancer are summarized in Tables I–III. In osteosarcoma, the median age at diagnosis was 20.5 years (range, nine to 68 years); 57.3% of the patients were males; 57.1% of cases were osteoblastic subtype, and 14.3% chondroblastic; 57.1% presented with metastasis at diagnosis, and 14.3% at follow-up; 71.4% exhibited relapse; with 53.6% patients at clinical stage 4 at diagnosis. In colorectal cancer, the median age at diagnosis was 58 years (range, 25–96 years); ~58% of the patients were male and for 21.5% of patients, the tumor was located in the colon. In the case of breast cancer, the median age at diagnosis was 52 years (range, 30–83 years); 74.2% of the patients were pregnant at <30 years of age; 50.5% of patients breast-fed for more than six months; and the majority of patients (67%) were of clinical stage 2 at diagnosis. The genotyping call rate was >90%. The call rate is the success rate for assigning genotypes; this implies that allele discrimination was optimal. Allele and genotype frequencies of Asn118Asn (*ERCC1)* and rs13181 (*ERCC2*) were distributed according to the HWE model in each group in all types of cancer (P>0.05). Nevertheless no association with any particular type of cancer was found for the aforementioned SNPs (Tables IV–VI). Notably, the HWE test for rs1799793 showed that the genotype frequencies deviated from expected values (P<0.05) and that this SNP was indeed associated with breast cancer [OR, 9.00; 95% confidence interval (CI), 1.10–73.50; P=0.01].

## Discussion

*ERCC1* and *ERCC2* genes participate in DNA repair and therefore, when mutated, may contribute to genome instability. Thus, genetic variants in these genes may be associated with the susceptibility of various types of cancer, such as osteosarcoma, and colorectal and breast cancer. *In vitro* studies have shown that rs11615 (*ERCC1*) is linked to reduced mRNA levels and a consequence reduction in protein production (17). By contrast, the rs13181 and rs1799793 SNPs of the *ERCC2* gene are associated with a deficient DNA repair capacity (18,19). Nonetheless, studies in different populations should be performed in order to confirm the association of the aforementioned variants and different types of cancer; since ethnicity is a crucial factor that could modify the penetrance of disease-associated genetic variants (20–22). The present data showed a positive association between rs1799793 and breast cancer, and a lack of association between the rest of the studied variants and osteosarcoma, and colorectal and breast cancer in the Mexican patients. This SNP has been studied in different populations and deviation from HWE has been observed in at least three populations (23–25). Thus it could be possible that this SNP, which is associated with cancer risk and may have a deleterious effect on survival and is a reason for a population to deviate from the expected frequencies of the HWE model. Although more and larger studies are required to confirm this association in different populations, to the best of our knowledge, the present study is the first attempt to analyze the genetic variants of *ERCC1* and *ERCC2* genes in Mexican-mestizo patients with osteosarcoma, and colorectal and breast cancer.

## Figures and Tables

**Table I:** Clinical data of osteosarcoma patients (n=28).

Clinical data	Value
Age at diagnosis, years	
Median	20.5
Range	9–68
Gender, %	
Female	42.9
Male	57.1
Subtype, %	
Osteoblastic	57.1
Chondroblastic	14.3
Other	28.6
Tumor location, %	
Femur	85.7
Tibia	3.6
Arm	10.7
Necrosis, %	
Good	70.0
Poor	30.0
Metastasis, %	
No	28.6
At diagnosis	57.1
At follow-up	14.3
Status, %	
Alive	85.7
Succumbed	14.3
Relapse, %	
No	71.4
Yes	28.6
Clinical stage at diagnosis, %	
2	17.8
3	28.6
4	53.6
Karnofsky Score, %	
80	21.4
70	28.6
60	35.7
50	3.6
40	10.7

**Table II:** Clinical data of colorectal cancer patients (n=108).

Clinical data	Value
Age at diagnosis, years	
Median	58
Range	25–96
Gender, %	
Female	42.0
Male	58.0
Tumor location, %	
Colon	54.1
Rectum	45.9

**Table III:** Clinical data of breast cancer patients (n=71).

Clinical data	Value
Age at diagnosis, years	
Median	52
Range	30–83
Pregnancy prior to 30 years of age, %	
No	25.8
Yes	74.2
Breastfeeding more than six months, %	
No	49.5
Yes	50.5
Stage, %	
1	12.4
2	67.0
3	20.6
HER2(+), %	
No	85.6
Yes	14.4

HER2, human epidermal growth factor receptor 2.

**Table IV:** Genotype frequencies of *ERCC1* and *ERCC2* polymorphisms of control group (n=97) and osteosarcoma patients (n=28).

Variants	Genotypes	Controls, n (%)	Patients, n (%)	OR (95% CI)	P-value^a^
*ERCC1* rs11615	GG	59 (60.8)	16 (57.2)	1.00	
	GA	32 (33.0)	9 (32.1)	1.04 (0.41–2.61)	0.94
	AA	6 (6.2)	3 (10.7)	1.84 (0.41–8.20)	0.42
	GG/GA	91 (93.8)	25 (89.3)	1.16 (0.50–2.73)	0.73
*ERCC2* rs13181	AA	64 (66.0)	21 (75.0)	1.00	
	AC	31 (31.9)	7 (25.0)	0.69 (0.26–1.79)	0.44
	CC	2 (2.1)	0 (0.0)	0.60 (0.03–12.99)	0.42
	AA/AC	95 (97.9)	28 (100.0)	0.65 (0.25–1.68)	0.37
*ERCC2* rs1799793	CC	68 (70.1)	21 (75.0)	1.00	
	CT	8 (8.3)	3 (10.7)	1.21 (0.29–4.99)	0.79
	TT	21 (21.6)	4 (14.3)	0.62 (0.19–1.99)	0.42
	CC/CT	76 (78.4)	24 (85.7)	0.78 (0.29–2.04)	0.61

a χ^2^ or Fisher’s exact test.

**Table V:** Genotype frequencies of *ERCC1* and *ERCC2* polymorphisms of control group (n=119) and colorectal cancer patients (n=108).

Variants	Genotypes	Controls, n (%)	Patients, n (%)	OR (95% CI)	P-value^a^
*ERCC1* rs11615	GG	58 (48.7)	46 (42.6)	1.00	
	GA	50 (42.1)	47 (43.5)	1.18 (0.68–2.06)	0.55
	AA	11 (9.2)	15 (13.9)	1.72 (0.72–4.10)	0.22
	GG/GA	108 (90.8)	93 (86.1)	1.28 (0.76–2.16)	0.35
*ERCC2* rs13181	AA	74 (62.2)	69 (63.9)	1.00	
	AC	39 (32.8)	33 (30.5)	0.91 (0.51–1.60)	0.74
	CC	6 (5.0)	6 (5.6)	1.07 (0.33–3.48)	0.91
	AA/AC	113 (95.0)	102 (94.4)	0.93 (0.54–1.59)	0.79
*ERCC2* rs1799793	CC	81 (68.1)	74 (68.5)	1.00	
	CT	23 (19.3)	26 (24.1)	1.24 (0.65–2.35)	0.52
	TT	15 (12.6)	8 (7.4)	0.58 (0.23–1.46)	0.24
	CC/CT	104 (87.4)	100 (92.6)	0.98 (0.56–1.71)	0.94

a χ^2^ or Fisher’s exact test.

**Table VI:** Genotype frequencies of *ERCC1* and *ERCC2* polymorphisms of control group (n=74) and breast cancer patients (n=71).

Variants	Genotypes	Controls, n (%)	Patients, n (%)	OR (95% CI)	P-value^a^
*ERCC1* rs11615	GG	40 (54.1)	38 (53.5)	1.00	
	GA	27 (36.5)	28 (39.4)	1.09 (0.55–2.18)	0.80
	AA	7 (9.4)	5 (7.1)	0.75 (0.22–2.57)	0.65
	GG/GA	67 (90.6)	66 (92.9)	1.02 (0.53–1.96)	0.95
*ERCC2* rs13181	AA	45 (60.8)	49 (69.1)	1.00	
	AC	27 (36.5)	19 (26.7)	0.65 (0.32–1.32)	0.23
	CC	2 (2.7)	3 (4.2)	1.38 (0.22–8.62)	0.73
	AA/AC	72 (97.3)	68 (95.8)	0.69 (0.35–1.38)	0.30
*ERCC2* rs1799793	CC	54 (72.9)	54 (76.0)	1.00	
	CT	1 (1.4)	9 (12.7)	9.00 (1.10–73.50)	0.01^b^
	TT	19 (25.7)	8 (12.3)	0.42 (0.17–1.04)	0.06
	CC/CT	55 (74.3)	63 (88.7)	0.85 (0.40–1.79)	0.67

a χ^2^ or Fisher’s exact test.

b Deviated from Hardy-Weinberg equilibrium.

## References

[1] Wood RD, Mitchell M, Sgouros J, Lindahl T (2001). Human DNA repair genes. Science.

[2] De Silva IU, McHugh PJ, Clingen PH, Hartley JA (2000). Defining the roles of nucleotide excision repair and recombination in the repair of DNA interstrand cross-links in mammalian cells. Mol Cell Biol.

[3] Chen ZP, Malapetsa A, McQuillan A (1997). Evidence for nucleotide excision repair as a modifying factor of O6-methylguanine-DNA methyltransferase-mediated innate chloroethylnitrosourea resistance in human tumor cell lines. Mol Pharmacol.

[4] Goode EL, Ulrich CM, Potter JD (2002). Polymorphisms in DNA repair genes and associations with cancer risk. Cancer Epidemiol Biomarkers Prev.

[5] Fontana L, Bosviel R, Delort L (2008). DNA repair gene ERCC2, XPC, XRCC1, XRCC3 polymorphisms and associations with bladder cancer risk in a french cohort. Anticancer Res.

[6] Huang MY, Fang WY, Lee SC, Cheng TL, Wang JY, Lin SR (2008). ERCC2 2251A>C genetic polymorphism was highly correlated with early relapse in high-risk stage II and stage III colorectal cancer patients: a preliminary study. BMC Cancer.

[7] Li J, Jin W, Chen Y, Di G, Wu J, Shao ZM (2008). Genetic polymorphisms in the DNA repair enzyme ERCC2 and breast tumour risk in a Chinese population. J Int Med Res.

[8] Bernard-Gallon D, Bosviel R, Delort L (2008). DNA repair gene ERCC2 polymorphisms and associations with breast and ovarian cancer risk. Mol Cancer.

[9] Yao L, Qiu LX, Yu L (2010). The association between ERCC2 Asp312Asn polymorphism and breast cancer risk: a meta-analysis involving 22,766 subjects. Breast Cancer Res Treat.

[10] Yang Z, Fang X, Pei X, Li H (2013). Polymorphisms in the ERCC1 and XPF genes and risk of breast cancer in a Chinese population. Genet Test Mol Biomarkers.

[11] Tan X, Wang Y, Shi L (2014). Polymorphism of ERCC2 Asp312Asn with lung cancer risk: evidence from 20,101 subjects. Genet Test Mol Biomarkers.

[12] Wen F, Zhao Z, Liu C (2014). A pooled analysis of the ERCC2 Asp312Asn polymorphism and esophageal cancer susceptibility. Tumour Biol.

[13] Deng Q, Sheng L, Su D (2011). Genetic polymorphisms in ATM, ERCC1, APE1 and iASPP genes and lung cancer risk in a population of southeast china. Med Oncol.

[14] Lin H, Lin D, Zheng C (2014). Association of XPD Lys751Gln polymorphism with head and neck cancer susceptibility: evidence from 11,443 subjects. Diagn Pathol.

[15] Zhang L, Wang J, Xu L (2012). Nucleotide excision repair gene ERCC1 polymorphisms contribute to cancer susceptibility: a meta-analysis. Mutagenesis.

[16] Gustincich S, Manfioletti G, Del Sal G, Schneider C, Carninci P (1991). A fast method for high-quality genomic DNA extraction from whole human blood. Biotechniques.

[17] Yu JJ, Lee KB, Mu C (2000). Comparison of two human ovarian carcinoma cell lines (A2780/CP70 and MCAS) that are equally resistant to platinum, but differ at codon 118 of the ERCC1 gene. Int J Oncol.

[18] Lunn RM, Helzlsouer KJ, Parshad R (2000). XPD polymorphisms: effects on DNA repair proficiency. Carcinogenesis.

[19] Duell EJ, Wiencke JK, Cheng TJ (2000). Polymorphisms in the DNA repair genes XRCC1 and ERCC2 and biomarkers of DNA damage in human blood mononuclear cells. Carcinogenesis.

[20] Lichtenstein P, Holm NV, Verkasalo PK (2000). Environmental and heritable factors in the causation of cancer - analyses of cohorts of twins from Sweden, Denmark, and Finland. N Engl J Med.

[21] Yin M, Yan J, Martinez-Balibrea E (2011). ERCC1 and ERCC2 polymorphisms predict clinical outcomes of oxaliplatin-based chemotherapies in gastric and colorectal cancer: a systemic review and meta-analysis. Clin Cancer Res.

[22] Adeyemo A, Rotimi C (2010). Genetic variants associated with complex human diseases show wide variation across multiple populations. Public Health Genomics.

[23] Paszkowska-Szczur K, Scott RJ, Gorski B (2014). Polymorphisms in nucleotide excision repair genes and susceptibility to colorectal cancer in the Polish population. Mol Biol Rep.

[24] Gao K, Mu SQ, Wu ZX (2014). Investigation of the effects of single-nucleotide polymorphisms in DNA repair genes on the risk of glioma. Genet Mol Res.

[25] Chu H, Gu D, Xu M (2013). A genetic variant in ERCC2 is associated with gastric cancer prognosis in a Chinese population. Mutagenesis.

